# Fibroblast Growth Factor 2 Causes G2/M Cell Cycle Arrest in Ras-Driven Tumor Cells through a Src-Dependent Pathway

**DOI:** 10.1371/journal.pone.0072582

**Published:** 2013-08-26

**Authors:** Jacqueline Salotti, Matheus H. Dias, Marianna M. Koga, Hugo A. Armelin

**Affiliations:** 1 Departamento de Bioquímica, Instituto de Química, Universidade de São Paulo, São Paulo, Brazil; 2 Instituto Butantan, CATcepid, São Paulo, Brazil; II Università di Napoli, Italy

## Abstract

We recently reported that paracrine Fibroblast Growth Factor 2 (FGF2) triggers senescence in Ras-driven Y1 and 3T3^Ras^ mouse malignant cell lines. Here, we show that although FGF2 activates mitogenic pathways in these Ras-dependent malignant cells, it can block cell proliferation and cause a G2/M arrest. These cytostatic effects of FGF2 are inhibited by PD173074, an FGF receptor (FGFR) inhibitor. To determine which downstream pathways are induced by FGF2, we tested specific inhibitors targeting mitogen-activated protein kinase (MEK), phosphatidylinositol 3 kinase (PI3K) and protein kinase C (PKC). We show that these classical mitogenic pathways do not mediate the cytostatic activity of FGF2. On the other hand, the inhibition of Src family kinases rescued Ras-dependent malignant cells from the G2/M irreversible arrest induced by FGF2. Taken together, these data indicate a growth factor-sensitive point in G2/M that likely involves FGFR/Ras/Src pathway activation in a MEK, PI3K and PKC independent manner.

## Introduction

The fibroblast growth factor (FGF) family currently comprises 22 distinct protein members in humans and mice. This family of signaling factors governs an expanding number of biological and pathological processes [1]. In particular, FGF2 (or basic FGF), the prototypical member [2], has important functions in development [3] and in the adult organism [4]. FGF2 promotes angiogenesis, proliferation, apoptosis, differentiation, wound healing, chemotaxis and motility of different cell types. Because of its angiogenic and mitogenic properties, FGF2 is also recognized as a potential oncoprotein [5] [6] [7] [8]. In addition, FGF2 can also act as an antiapoptotic factor, rendering tumor cells more resistant to chemotherapy [9]. On the other hand, some researchers have reported that FGF2 can suppress proliferation by a variety of mechanisms, such as apoptosis in chondrocytes [10], p53-independent cell death in Ewing’s sarcoma tumors [11] [12], G1 arrest in MCF-7 human breast cancer cells, rat chondrosarcoma and pituitary lactotroph GH4 cells [13]–[16] and G2 arrest in a human neuroepithelioma cell line [17]. In addition, our laboratory recently reported that exogenous recombinant FGF2 irreversibly inhibits the proliferation of Ras-dependent malignant mouse cells but not immortalized nontumorigenic cell lines [18]. These observations led us to hypothesize that the FGF2/FGFR signaling system could initiate novel tumor-defense pathways in Ras-dependent malignant cells.

The binding of FGF2 to the high affinity cell surface FGF-Receptors (FGFRs) and to heparan sulfate proteoglycans (HSPGs) leads to the formation of a ternary complex between FGFR, FGF and HSPG [19], which initiates multiple intracellular signaling cascades [20]. Five FGFRs have been described, FGFR1 to FGFR5 [21]–[24]. As a general rule, the structure of FGFRs is comprised of an extracellular ligand-binding region, which can contain two or three immunoglobulin-like loops (IgI, IgII, IgIII domains), a single transmembrane domain, and two intracellular tyrosine-kinase domains (FGFR5 lacks this kinase domain) [19], [20]. There are several types of FGFs, guiding different effects in distinct target cells. In order to reach this kind of diversity, the FGF signaling system demands a variation in the FGFRs, which is achieved through a splicing event that occurs in IgIII [25]–[27]. The IgIII domain of FGFR1 to FGFR3 is encoded by the invariant exon IIIa followed by one of two alternative spliced exons: IIIb or IIIc (referred to as isoforms FGFRIIIb, FGFRIIIc). These FGFRs isoforms generated by alternative splicing have been shown to be important in determining FGFs binding specificity and are expressed according to cell type: epithelial cells contain FGFRIIIb isoforms, whereas mesenchymal cells contain FGFRIIIc isoforms [28]. Besides that, FGFs that bind to FGFRIIIb are often released by mesenchymal cells, whereas FGFs that bind to FGFRIIIc are released by epithelial cells, establishing a paracrine system in epithelia-mesenchyma communication, which is crucial to normal development and tissue homeostasis. Moreover, deregulation in this signaling system can promote mesenchymal-to-epithelial transition in tumor cells [29], [30].

The Ras/Raf/MEK/extracellular signal-regulated kinase (ERK) cascade couples signals from cell surface receptors to transcription factors, which regulate gene expression of proteins that control cell cycle progression [31]. Activating mutations in *Ras* genes are very common in the development of tumors and found in 20–25% of all human cancers [32]. The sustained activation of ERK 1/2 in G1 promotes S phase entry whereas the transient activation of ERK does not [33]. On the other hand, high intensity ERK signal causes growth arrest through p21/Cdkn1a induction [34], [35]. Thus, the biological outcome in response to ERK signaling will depend on a combination of stimulation nature, intensity and duration.

Growth factor signaling can also activate the PI3K/Akt and PKC pathways. Akt activation increases glucose uptake, glycolysis, glycogen synthesis, protein synthesis, cell size, cell cycle progression and anti-apoptotic responses [36]. PKC serine/threonine kinases are activated by signals such as intracellular increases in diacylglycerol (DAG) or calcium ions (Ca^2+^). PKC enzymes play important roles in several signal transduction cascades involved in cell-cycle regulation, cellular survival, malignant transformation and apoptosis [37].

The Src family of protein tyrosine kinases has been linked to a large number of human malignancies. Elevated levels of Src activity, the first and better known member of this family, are related to aggressiveness and poor prognosis [38]. Src overactivity drives tumor progression by promoting cell survival and proliferation, motility, invasion, and also angiogenesis [39]. On the other hand, it was recently shown that even in malignant cells, Src overactivity above a threshold can lead to cell death instead of growth advantages [40]. In addition, Src play an important role in FGFR1 activation, transport, and signaling dynamics through directly interaction with activated FGFR1 complexes [41].

In the present study, we analyzed the cellular and molecular mechanisms of paracrine FGF2 in Ras-dependent Y1 and 3T3^Ras^ mouse malignant cell lines. Our purpose was to explore the hypothesis that tyrosine kinase receptors, here typified by FGFRs, can initiate a ligand-dependent response in malignant cells, triggering tumor defense mechanisms. To test this hypothesis, we have mainly focused on the Y1 cell line, which carries *Kras* oncogene amplification [42] but can be controlled at the G0/G1 → S cell cycle transition. Specifically, in Y1 cells, the Ras/ERK pathway is strictly dependent on activation by serum factors and FGF2. Because of these phenotypic features, Y1 cells were used to study the mechanisms underlying FGF2’s antagonistic actions, namely, its classical mitogenic activity and the novel cytostatic function. In addition, we examined 3T3^Ras^, a stable subline derived from an H-Ras^V12^ transfection of Balb 3T3 fibroblasts [43]. These cells are tumorigenic but are also sensitive to FGF2 treatment [18].

As FGF2/FGFR activates various signaling pathways such as MEK/ERK, PI3K/Akt, PLC/PKC and Src, we investigated whether the cytostatic effect of FGF2 involves these canonical pathways. Our herein reported results suggested that, in K-Ras-driven Y1 malignant cells, FGF2 activates the antiproliferative pathway FGF2/FGFR → Src → RhoA to irreversibly block the cell cycle G2 → M transition independently from PI3K/Akt, Ras/ERK and PLCγ/PKC mitogenic pathways.

## Results

### FGF2 Inhibits the Proliferation of Ras-driven Malignant Cells Through FGFR Tyrosine Kinase Activation

**Figure 1:**
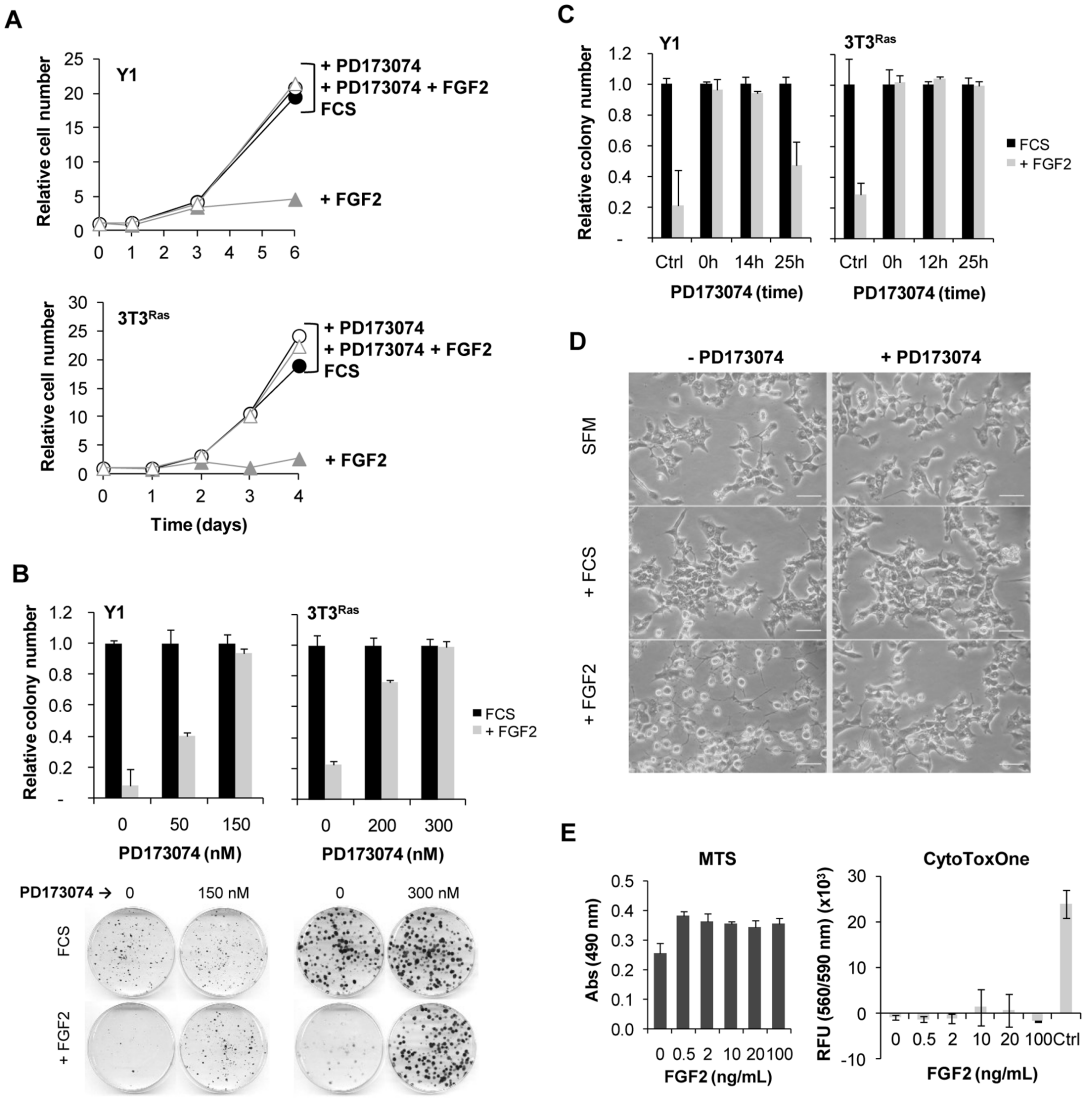
FGF2-induced cell growth inhibition of Y1 and 3T3^Ras^ cells is dependent on FGF receptor activation. (**A**) Growth curves with FGF2 (10 ng/mL) and PD173074 (150 nM for Y1 and or 300 nM for 3T3^Ras^ cells) added 5 minutes before FGF2 (10^4^ cells/cm^2^ were plated). (**B**) Clonogenic assays of Y1 and 3T3^Ras^ cells treated with FGF2 and/or PD173074 (added 5 minutes before FGF2) for 24 hours. Inset below shows one representative clonogenic assay. (**C**) Clonogenic assays of Y1 and 3T3^Ras^ cells in the presence of FGF2, added at time 0, and PD173074, added after FGF2 at the indicated times. (**D**) Microphotographs for morphological analysis of Y1 cells treated with FCS (10%) or FGF2 (10 ng/mL) for 48 hours in the presence, where indicated, of PD173074 (150 nM). Scale bar = 50 µm. (**E**) MTS Assay (CellTiter 96® AQueous) and CytoTox-One™ Assay (membrane integrity) performed with increasing concentrations of FGF2. RFU, Relative Fluorescent Unit. Control refers to 100% cell lysis. Values are mean ± s.e.m (n = 2–5). FCS, fetal calf serum.

FGF2 strongly inhibited the population growth and colony formation of Y1 and 3T3^Ras^ tumor cells (Figures 1A and 1B, respectively; compare FCS and +FGF2 conditions). To probe into mechanisms of FGF2’s inhibitory effects, we first addressed whether FGFR tyrosine kinase activation mediates these effects of FGF2 by using PD173074, a selective pan-inhibitor of the tyrosine kinase activity of FGFR [44]. In Figures 1A and 1B, we show that the FGFR inhibitor PD173074 fully blocked FGF2’s inhibitory effects by restoring population and clonogenic growth of Y1 and 3T3^Ras^ tumor cells. The minimal PD173074 concentration required to abolish FGF2 is 150 nM for Y1 cells and 300 nM for 3T3^Ras^ cells. In colony formation assays, PD173074 restored cell survival even when added 14 or 25 hours after FGF2 addition to Y1 and 3T3^Ras^ cultures, respectively (Figure 1C). These results suggest that FGF2 can cause multiple negative effects throughout the cell cycle.

In addition, we also observed that FGF2 induced morphological alterations where cells become round and refringent (Figure 1D, left panel), which were also blocked by PD173074 (Figure 1D, right panel). To further investigate the FGF2-induced cell proliferation inhibition, we analyzed the cellular metabolic rate (MTS assay) and the membrane integrity. In the presence of FGF2, cells remained metabolically active and kept membrane’s integrity (Figure 1E). Thus, FGF2 induces a cytostatic effect involving cytoskeletal alterations, but without triggering apoptosis or necrotic cell death. These conclusions are in agreement with our previously published results and interpretations, which reported a type of cellular senescence induced by FGF2 [18].

**Figure 2:**
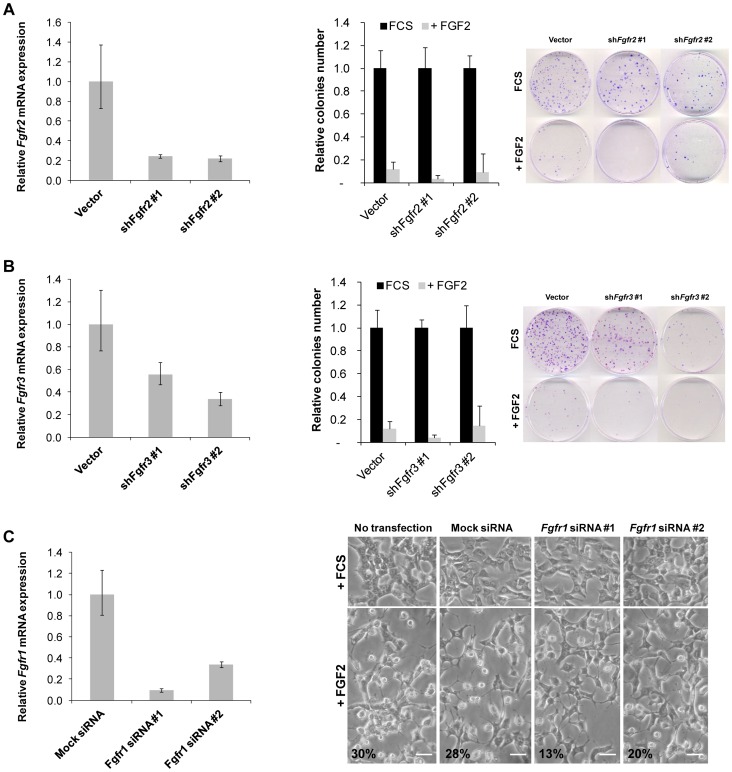
Knockdown of FGFR1, but not FGFR2 nor FGFR3, reduces FGF2’s cytostatic effects in Y1 cells. (**A**) Panel on the left shows the knockdown efficiency of FGFR2 in two independent sublines. Panels on the right are clonogenic assays in sublines with FGFR2 knockdown in the presence of FGF2 (10 ng/mL) for 24 h. Inset on the right shows one representative example of clonogenic assay in the indicated conditions. (**B**) Panel on the left shows the knockdown efficiency of FGFR3 in two independent sublines. Panels on the right are clonogenic assays in sublines with FGFR3 knockdown in the presence of FGF2 (10 ng/mL) for 24 h. Inset on the right shows one representative example of clonogenic assay in the indicated conditions. (**C**) Panel on the left shows the knockdown efficiency of FGFR1 by two independent siRNAs sequences. Panels on the right are representative microphotographs for morphological analysis of FGFR1 knockdown under FCS or FGF2 stimulation for 24 h. Scale bar = 50 µm. Values are mean ± s.e.m (n = 3). FCS, fetal calf serum.

Next we characterized the FGFRs present in Y1 cells to further approach the FGFR specificity by RNAi. We found that Y1 cells express FGFR1IIIc, FGFR2IIIc, FGFR3IIIc and FGFR5 (Figure S1). Since FGFR5 lacks the tyrosine kinase domain, we generated sh*Fgfr2* and sh*Fgfr3* stable clonal cell lines, which displayed downregulated FGFR2 and FGFR3, respectively (Figures 2A and 2B, left panels). However, these sh*Fgfr2* and sh*Fgfr3* cell lines were still susceptible to inhibition of clonogenic cell growth (Figures 2A and 2B, right panels), implying that FGFR2 and FGFR3 are not important mediators of FGF2’s cytostatic effects. Unfortunately, out of 18 sh*Fgfr1* clonal cell lines analyzed, none presented low levels of FGFR1 expression (not shown). To overcome this technical limitation, we transiently knocked down FGFR1 by siRNA and observed that cells with low levels of FGFR1 were resistant to the morphological alterations induced by FGF2 (Figure 2C), suggesting that FGFR1 is the main receptor triggering FGF2’s cytostatic effects in Y1 cells.

### FGF2 Activates Mitogenic ERK 1/2 but also Triggers an Antimitotic Process that Later Inhibits the DNA Synthesis Induced by Serum

**Figure 3:**
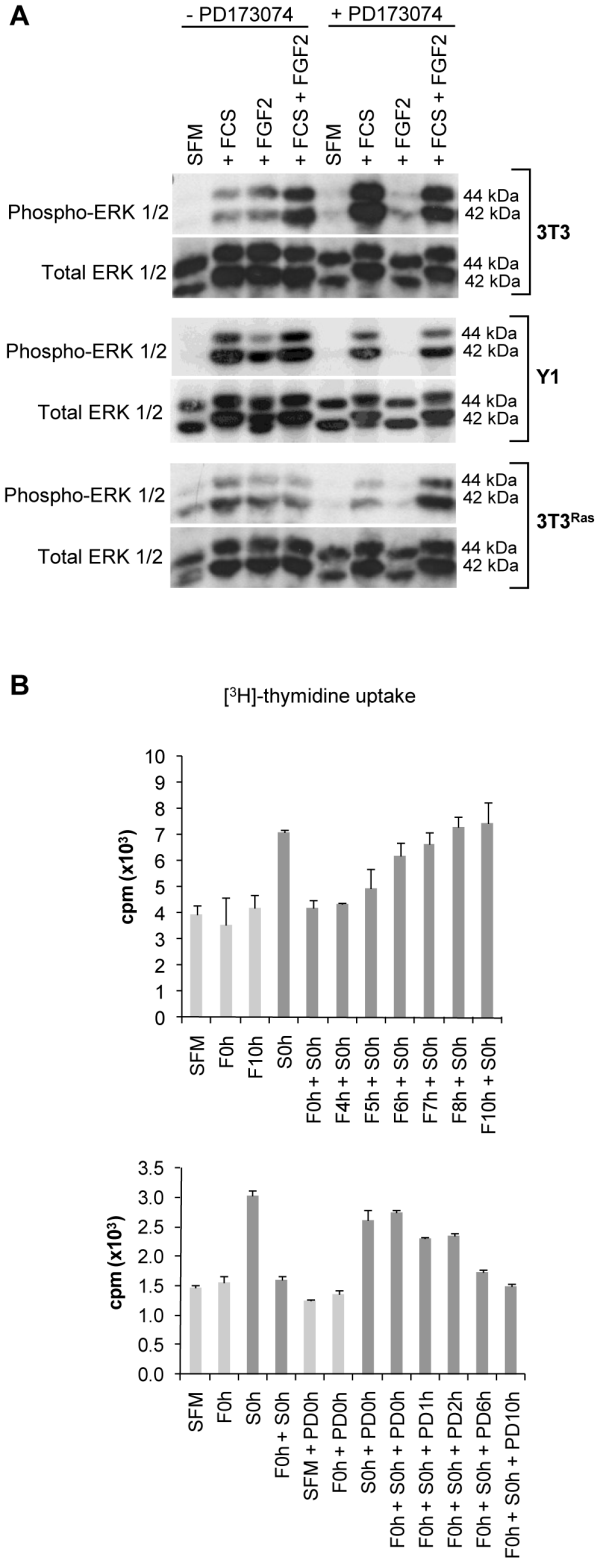
(A) FGF2 exerts its classical mitogenic activity via FGF receptor tyrosine kinase activation. Balb 3T3, Y1, and 3T3^Ras^ cells were synchronized in G0/G1 by serum starvation and stimulated with FCS (10%) and/or FGF2 (10 ng/mL) for 5–15 minutes in the presence of PD173074 (150 nM). Data are representative of two independent experiments. Phospho-ERK1/2 (phosphorylated at Thr202/Tyr204) was analyzed by immunoblot. Total ERK1/2 was used as a loading control. (**B**) DNA synthesis stimulated by serum is inhibited in the presence of FGF2. Upper panel: G0/G1-arrested Y1 cells were stimulated with FCS and/or FGF2 for 12 hours. Cells were pulse-labeled for 1 hour with [^3^H]-thymidine before harvesting, and the amount of incorporated radioactivity was measured. FCS was added at time 0, and FGF2 was added 0–10 hours after FCS stimulation. Lower panel: PD173074 was added at the indicated times (0–10 h) after stimulation with FCS and/or FGF2, both added at time 0. [^3^H]-thymidine incorporation results are presented as the mean ± s.e.m. (n = 2). SFM, serum-free media; FCS, fetal calf serum.

In immortalized nontumorigenic Balb 3T3 cells and malignant Y1 cells, but not in malignant 3T3^Ras^ cells, serum starvation causes G0/G1 arrest, decreasing phosphorylated ERK1/2 to negligible levels (Figure 3A, first lane, serum-free media or SFM). Despite this difference between cell lines in ERK regulation, FGF2 per se, like serum, activates ERK1/2 in all three cell lines (Figure 3A). In each case, FGF2 activation of ERK1/2 is blocked by the PD173074 inhibitor, which does not interfere with serum activity. FGF2 has been used for years as a classical mitogen, stimulating S phase entry in G0/G1-arrested Y1 malignant cells [45] and 3T3 immortalized fibroblasts [2]. Therefore, because PD173074 completely inhibits the ERK1/2 phosphorylation induced by FGF2 but not by serum (Figure 3A), the mitogenic signal triggered by FGF2 is mediated by FGFR.

To better understand the effects of FGF2 on the G0/G1 → S transition, we analyzed S phase entry by [^3^H]-thymidine uptake into DNA. In G0/G1-arrested Y1 cells stimulated by serum, previous results of [^3^H]-thymidine pulse labeling kinetics have shown that DNA synthesis started to increase by 8–9 hours (end of G1 phase) and picked by 11–12 hours (middle of S phase) [18], [45]. Thus, at time 0, we stimulated G0/G1-arrested Y1 cells with serum and incorporated [^3^H]-thymidine between 11–12 hours. DNA synthesis stimulated by serum was completely abolished by FGF2 that was concomitantly added at time 0 (Figure 3B, upper panel). However, the FGF2 inhibition of serum-stimulated DNA synthesis was completely eliminated when PD173074 was added to culture media within the first two hours after FGF2 addition (Figure 3B, lower panel). Furthermore, FGF2 coherently inhibited the DNA synthesis stimulated by serum only when added within the first five hours of serum stimulation (Figure 3B, upper panel). Altogether these results suggest that, in early G1 phase, the FGF2/FGFR signaling axis concomitantly triggers two antagonistic pathways: first, the canonical ERK mitogenic pathway (Figure 3A) and, second, a parallel unsuspected novel anti-proliferative pathway, which cause a late inhibition of serum induced DNA synthesis (Figure 3B). In addition to this conclusion, it is important to highlight that untreated control cells maintained in serum-free medium displayed relatively high background levels of DNA synthesis that does not change in the presence of FGF2 and/or PD173074, indicating partial deregulation of the quiescence-proliferation switch in Y1 cells.

### FGF2 Delays Progression Through S Phase and Irreversibly Arrests Cells in the G2/M Interface of the Cell Cycle

**Figure 4:**
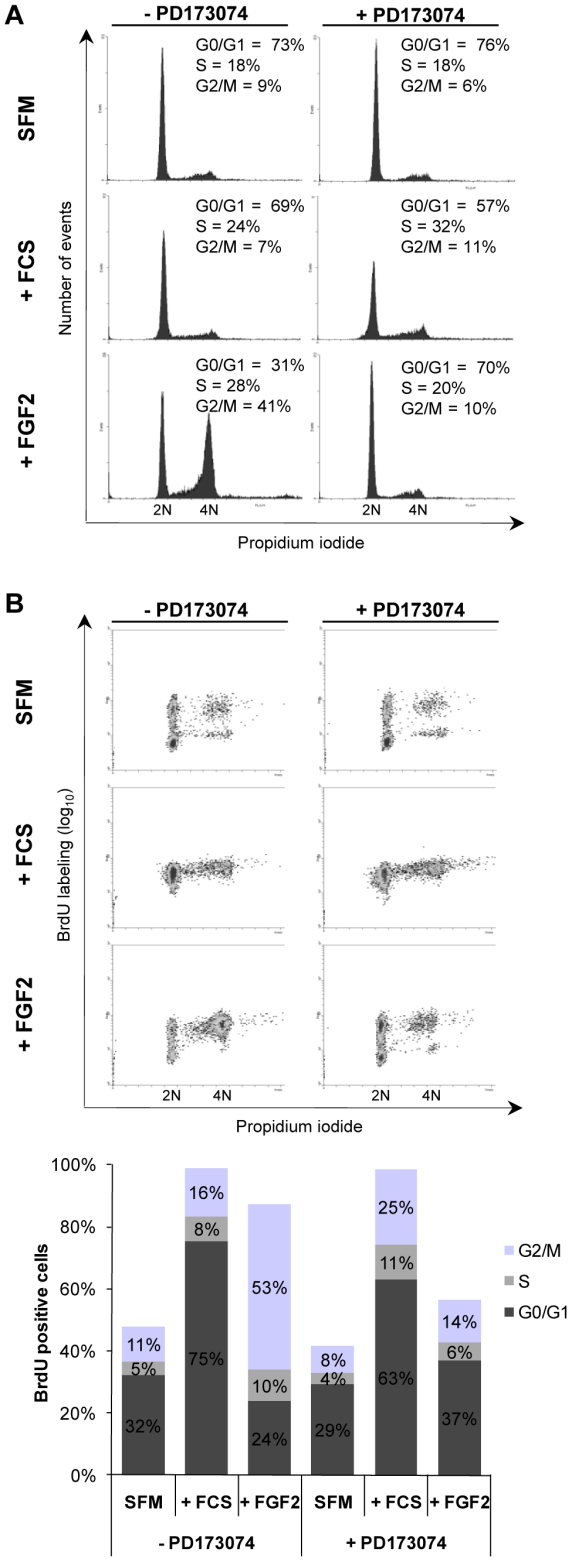
FGF2 causes G2/M arrest in Y1 cells. Flow cytometry histograms show DNA content in Y1 cells after 48 hours of FCS (10%) and FGF2 (10 ng/mL) treatment in G0/G1-starved cells. Cells were treated at time 0 and stained for DNA content and BrdU uptake. (**A**) Quantification of G0/G1, S and G2/M phases was based on DNA content. (**B**) Quantification of G0/G1, S and G2/M phases was based on DNA content versus BrdU labeling. Quantification of cell cycle phases was gated from the 2N to the 4N population only. Approximately 10^4^ cells were analyzed. SFM, serum-free media; FCS, fetal calf serum.

To further analyze the effects of FGF2 on cell cycle progression, G0/G1-arrested Y1 cells were stimulated with serum and FGF2 for 48 hours and then analyzed by flow cytometry after BrdU sustained labeling and propidium iodide staining. G0/G1-arrested cells in SFM were not completely quiescent; some cycled once irrespective of PD173074 presence (Figure 4A and 4B), in agreement with our [^3^H]-thymidine pulse labeling results (Figure 3B). As expected, all cells cycled at least once under serum stimulation (Figure 4B, lower panel). In addition, these results of long term experiments demonstrate that FGF2 per se had strong mitogenic activity because the large majority (87%) of FGF2-stimulated cells were labeled by BrdU, however 63% of the BrdU labeled cells did not complete cell division, being arrested in G2/M interface (Figure 4B, lower panel, FGF2 treatment). When PD173074 was added together with the FGF2 stimulus, it abolished both effects of FGF2, namely, the mitogenic and the anti-proliferative effects (Figure 4B, FGF2+PD); but it did not interfere with serum mitogenic effects (Figure 4B, FCS +/− PD). These results were confirmed and further detailed in a kinetics experiment: G0/G1-arrested Y1 cells were stimulated by serum or FGF2, sustained labeled with BrdU and followed for the next 12, 24, 36, 48 or 60 hours (Figure S2A). The majority of cells stimulated by serum entered S phase within 12 hours and cycled at least twice in the following 48 hours. On the other hand, FGF2-stimulated cells experienced a delayed entry into S phase and accumulated in G2/M interface (Figure S2A and S2C). Again, PD173074 abolished both mitogenic and inhibitory effects of FGF2 (Figure S2B and S2C). We conclude that 1) G0/G1-arrested Y1 cells present a leak in the quiescence to proliferation transition, in spite of displaying negligible levels of phosphorylated ERK1/2; and 2) FGF2 strongly stimulates the G1 → S transition but irreversibly restrains the G2 → M transition. Thus, the FGF2/FGFR signaling axis triggers two antagonistic cell cycle regulatory pathways in Ras-driven malignant cells: the classical mitogenic ERK pathway and a novel G2/M cell cycle arrest pathway.

### The Cytostatic Effect of FGF2 is Independent of the MEK/ERK, PI3K/AKT and PKC Pathways

**Figure 5:**
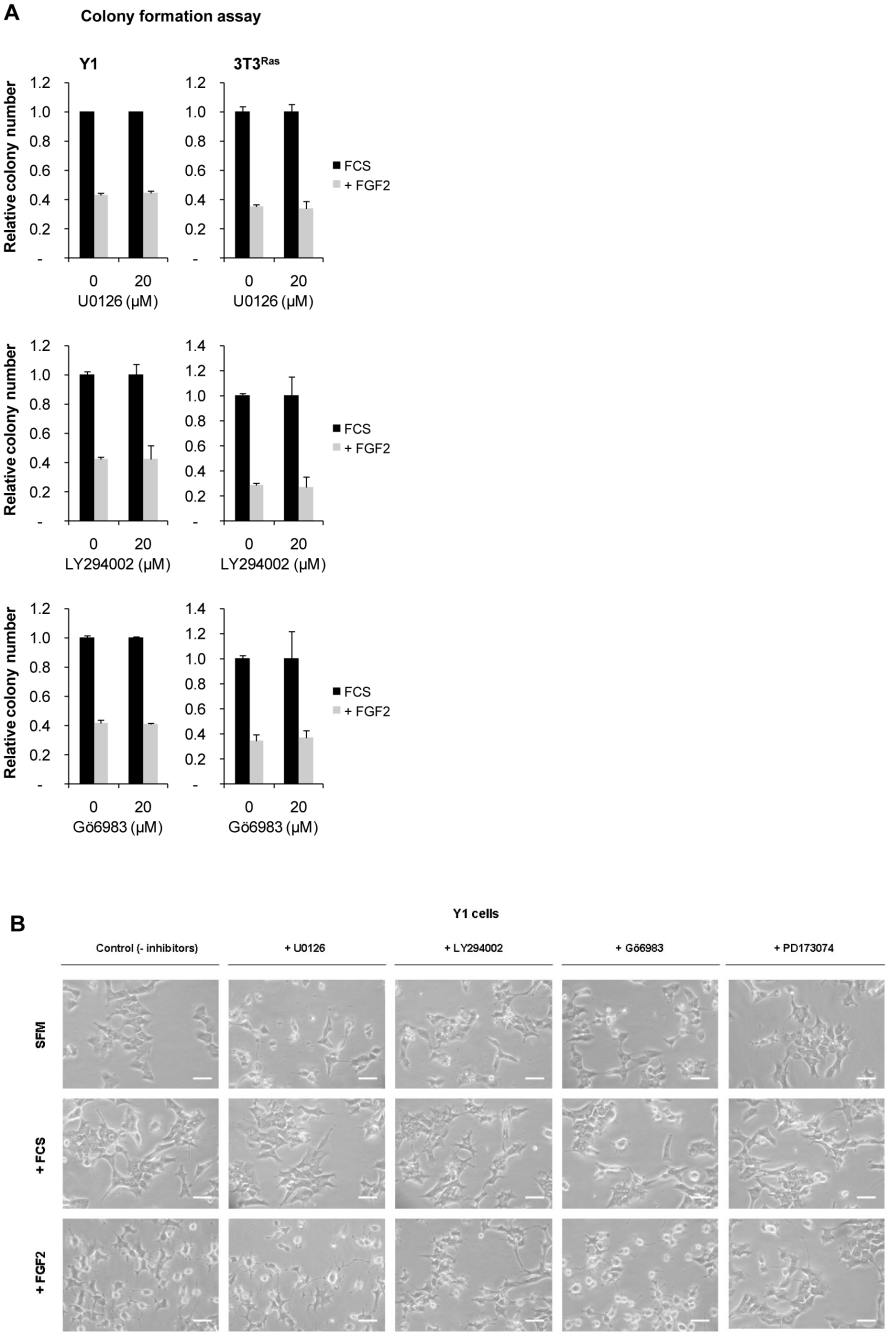
FGF2’s cytostatic mechanisms do not involve MEK, PI3K and PKC pathways. (**A**) Clonogenic assays of Y1 and 3T3^Ras^ cells treated with FGF2 and/or U0126, LY294002 or Gö6983 inhibitors (added 1 hour before FGF2) for 24 hours. Values are mean ± s.e.m. (n = 2). (**B**) Microphotographs for morphological analysis of Y1 cells under serum or FGF2 stimulation for 24 h, in the presence or absence of inhibitors: U0126, LY294002, Gö6983 or PD173074, added 1 h before the stimulation with serum or FGF2. The FGF receptor inhibitor (PD173074) was used as a positive control. Scale bar = 50 µm. FCS, fetal calf serum.

The mitotic and survival pathways known to be activated by FGFRs (MEK/ERK, PI3K/AKT and PKC) were next tested for possible involvement in the cytostatic effects of FGF2. In Y1 cells, but not in 3T3^Ras^ cells, ERK1/2 activation is strictly dependent on stimulation by mitogens (Figure 3A). In spite of this difference, FGF2 stimulates ERK1/2 activation in both cell lines (Figure 3A). Since, ERK1/2 are substrates of MEK1/2, we tested the MEK inhibitor U0126 [46]. U0126 did not protect cells from FGF2’s cytostatic effects as shown by clonogenic assays with both Y1 and 3T3^Ras^ cell lines (Figure 5A, upper panels) and morphological analysis (Figure 5B, plus U0126). Hence, U0126 treatment clearly uncoupled the mitogenic pathway MEK1/2 → ERK1/2 from the cytostatic pathways triggered by FGF2 in Ras-dependent malignant cells.

In Y1 cells, high constitutive levels of K-Ras-GTP maintain chronic basal levels of phosphorylated Akt, which are completely abolished by the PI3K inhibitors LY294002 and Wortmannin [47]. However, neither LY294002 nor Wortmannin (data not shown) blocks FGF2’s cytostatic effects, as shown by clonogenic assays with both Y1 and 3T3^Ras^ cell lines (Figure 5A, middle panels) and morphological analysis (Figure 5B, plus LY294002). Therefore, similar to MEK inhibition, PI3K inhibition uncouples the survival and mitogenic pathway PI3K → AKT from the FGF2-induced cytostatic mechanisms.

Similarly, the PKC inhibitor Gö6983 [48] does not block FGF2’s cytostatic activity as judged by clonogenic assays (Figure 5A, lower panels) and morphological analyses (Figure 5B, plus Gö6983), suggesting that the PKC pathway is not involved in the cytostatic effects of FGF2.

### The Cytostatic Effect of FGF2 is Dependent on Src Activity in a Complex Manner

**Figure 6:**
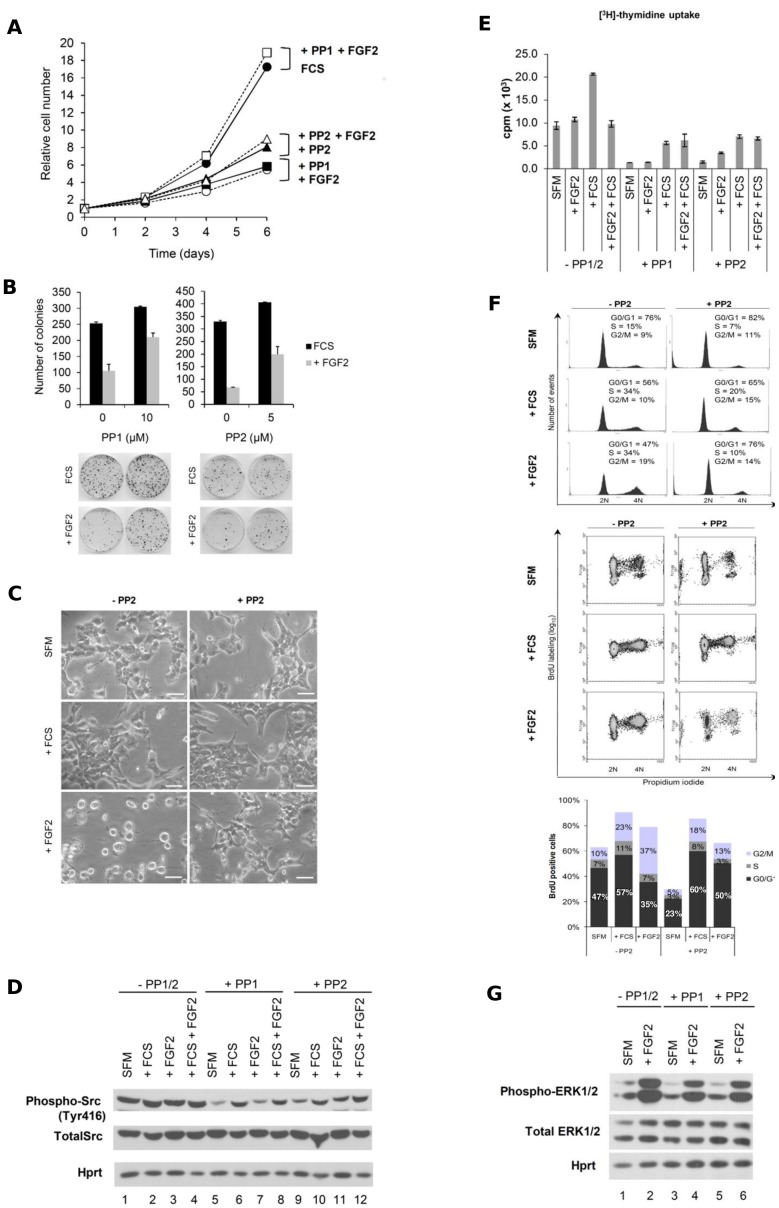
Src activation is necessary for FGF2’s cytostatic mechanisms. (**A**) Growth curves with FGF2 (10 ng/mL) and PP1 or PP2 (5 µM) added 1 hour before FGF2 (10^4^ cells/cm^2^ were plated). (**B**) Clonogenic assays of Y1 cells treated with FGF2 and/or PP1 (10 µM) or PP2 (5 µM) (added 30 minutes before FGF2) for 24 hours. Insets on the bottom show one representative clonogenic assay in the indicated conditions. Values are mean ± s.e.m. (n = 3). (**C**) Microphotographs for morphological analysis of Y1 cells under FCS or FGF2 stimulation for 48 hours, in the presence, where indicated, of PP2 (10 µM). (**D**) Y1 cells were synchronized in G0/G1 by serum starvation and stimulated with FCS (10%) and/or FGF2 (10 ng/mL) for 30 minutes in the presence of PP1 or PP2 (10 µM). Data are representative of four independent experiments. Phospho-Src (phosphorylated at Tyr416) and total Src were analyzed by immunoblot. Hprt was used as a loading control. (**E**) G0/G1-arrested Y1 cells were stimulated with FCS and/or FGF2 for 12 hours. Cells were pulse-labeled for 1 hour with [^3^H]-thymidine before harvesting, and the amount of incorporated radioactivity was measured. FCS and FGF2 were added at time 0. PP1 or PP2 were added 1 hour before stimulation with FCS and/or FGF2. [^3^H]-thymidine incorporation results are presented as the mean ± s.e.m. (n = 2). (**F**) Flow cytometry (FC) histograms show DNA content in Y1 cells after 24 and 48 hours of FCS and FGF2 treatment in G0/G1-starved cells. Cells were treated at time 0 and stained for DNA content and BrdU. FC histograms of DNA content (upper panels) and DNA/BrdU scatterplots (lower panels) of Y1 cells with or without PP2 (- PP2 and+PP2, respectively). Cells were stained with propidium iodide to assess DNA content. Biparametric flow cytometry analysis of DNA content and BrdU incorporation was performed on the same samples as described in the text. BrdU-positive cells were quantified by gates in the BrdU/DNA scatterplots. The upper panel shows the quantification of G0/G1, S and G2/M phases based on DNA content. The lower panel shows the quantification of G0/G1, S and G2/M phases based on DNA content versus BrdU labeling. Quantification of cell cycle phases, gated from the 2N to the 4N population only. Approximately 2×10^4^ cells were analyzed. (**G**) Y1 cells were synchronized in G0/G1 by serum starvation and stimulated with FCS (10%) and/or FGF2 (10 ng/mL) for 30 minutes in the presence of PP1 or PP2 (10 µM). Data are representative of two independent experiments. phospho-ERK1/2 (phosphorylated at Thr202/Tyr204) and total ERK 1/2 were analyzed by immunoblot. Hprt was used as a loading control. Scale bar = 50 µm. SFM, serum-free media; FCS, fetal calf serum.

The Src family of protein tyrosine kinases is normally maintained in an inactive state, but they can be transiently activated by growth factors stimuli [39]. We probed Src kinases involvement in FGF2’s cytostatic mechanisms in Y1 tumorigenic cells with the Src family selective inhibitors PP1 and PP2 [49]. The effects of Src inhibitors PP1 and PP2 on proliferation of Y1 mass cultures, Y1 clonogenic growth and DNA synthesis in G0/G1-arrested Y1 cells were somewhat complex. In Y1 mass cultures growing with 10% serum, sustained treatment with either FGF2 or PP1 severely inhibited cell proliferation; however, paradoxically, sustained treatment with FGF2 plus PP1 promoted optimal rate of cell proliferation (Figure 6A). Under the same conditions, PP2 displayed a similar qualitative pattern of effects (Figure 6A). On the other hand, in clonogenic growth assays, 24 hours of PP1 treatment significantly increased the number of colonies rescued after 10 days of growth in serum; in addition, 24 hours of PP1 treatment abolished the negative effect of FGF2 on clonogenic growth (Figure 6B). Furthermore, sustained PP2 treatment eliminated cell morphological alterations caused by 48 hours of FGF2 treatment (Figure 6C). Thus, in the short term, PP1 and PP2 promote cell survival, as suggested by clonogenic assays (Figure 6B), whereas in the long term, both PP1 and PP2 strongly limit rate of cell proliferation, as shown by growth curves (Figure 6A).

In the absence of serum factors, G0/G1-arrested Y1 cells display high basal levels of phosphorylated Src, which are reduced upon treatment of PP1 and PP2 (Figure 6D, compare lanes 1, 5 and 9). Moreover, in the same conditions, PP1 and PP2 drastically reduced basal levels of DNA synthesis as shown by ^3^H-thymidine pulse labeling (Figure 6E, compare SFM, minus and plus PP1 or PP2). Similar results were seen with 24 hours of BrdU sustained labeling (Figure 6F, compare SFM, minus and plus PP2). These observations were particularly noteworthy considering that U0126 and LY294002, inhibitors of MEK and PI3K respectively, caused small reductions in basal levels of DNA synthesis in G0/G1-arrested Y1 cells (not shown).

Src activation by serum and/or FGF2 is reduced upon treatment of PP1 and PP2 (Figure 6D). Furthermore, maximal levels of DNA synthesis induced by 12 hours of serum treatment were severely limited by PP1 and PP2, as shown by ^3^H-thymidine pulse labeling (Figure 6E). In addition, under these conditions, FGF2 did not stimulate DNA synthesis nor inhibited DNA synthesis stimulation by serum (Figure 6E). However, BrdU sustained labeling indicated that FGF2 per se, in the presence of PP2, stimulated both DNA synthesis and cell division (Figure 6F). These last results are in perfect agreement with the fact that 30 minutes of FGF2 treatment, in the presence or absence of PP1 and PP2, strongly activated the ERK mitogenic pathway (Figure 6G). Thus, the inhibition of Src abolished the G2/M arrest triggered by FGF2 without interfering with FGF2’s mitogenic activity.

## Discussion

We report here that the FGF2/FGFR signaling axis triggers antagonistic cell cycle regulatory pathways in Ras-driven Y1 malignant cells: 1) canonical mitogenic pathways, such as Ras/ERK, and 2) a novel G2/M cell cycle arrest pathway, which depends on Src’s deregulated constitutive activity. These FGF2’s effects point to unsuspected vulnerabilities in cell cycle homeostasis of Ras-dependent malignancies, which are quite common among human cancers. Malignant molecular signaling networks result from discrete deregulatory alterations in normal signaling systems. However, the possibilities of malignant signaling deregulations are likely to be severely constrained; the resulted malignant cell must yield a robust proliferating clonal subline that is sufficiently capable of initiating tumor growth. This notion supports the assumption that uncovering molecular deregulatory alterations underlying malignant transformation is not a scientifically unattainable endeavor. In this regard, the K-Ras-dependent Y1 cell line has been a unique experimental model for probing into critical molecular alterations of the Ras-dependent malignant network downstream of the FGF2/FGFR signaling axis [18].

The present reported results demonstrate that FGF2 inhibits cellular growth and colony formation in a PD173074 time- and dose-sensitive manner (Figure 1). Viability assays demonstrated that cells remain intact and metabolically active within 24 hours of exposure to FGF2, but they do exhibit morphological alterations suggestive of cellular stress (Figure 1). The morphological changes caused by FGF2 are exacerbated and distinct from the cytoskeletal reorganization induced by serum stimulation. In addition, the pattern of [^3^H]-thymidine uptake into DNA suggests that FGF2 triggers an antimitogenic process within the first hours of G1, where it transiently blocks DNA synthesis despite concurrent activation of ERK1/2 (Figure 3). Moreover, flow cytometry and BrdU analyses demonstrated that FGF2 promotes the G0/G1 → S → G2 transitions (mitogenic activity) but irreversibly arrests the progression from G2 → M (antimitogenic activity) (Figure 4). We further verified that PD173074 completely blocks FGF2’s effects, showing that both mitogenic and antimitogenic activities begin with the FGFR tyrosine kinase. However, specific inhibitors of MEK/ERK, PI3K/AKT and PKC signaling pathways (all mitogenic and downstream of FGFRs) do not block FGF2’s deleterious effects, suggesting that these pathways do not participate in the cytostatic molecular mechanisms triggered by FGF2 (Figure 5). On the other hand, in Y1 cells, Src tyrosine kinases inhibitors PP1 and PP2 interacted with FGF2 in a complex manner, implicating Src’s deregulated activity in FGF2’s cytostatic activity (Figure 6).

The PP1 Src inhibitor blocked Y1 cell proliferation as much as FGF2 did (Figure 6A); however, the combination of both PP1 and FGF2 strikingly stimulated optimal proliferation of Y1 cells (Figure 6A). On the other hand, PP1 and PP2 addition surprisingly promoted Y1 cell survival and clonal viability during the first 24 hours after single cell plating in clonogenic assays (Figure 6B). Y1 cells displayed high basal deregulated levels of active Src, which were further increased by FCS and/or FGF2 (Figure 6D). Altogether these results suggest that: 1) high chronic levels of deregulated active Src keep Y1 cells in the brink of oncogene stress; 2) reduction of deregulated active Src by 24 hours treatment with PP1 and PP2 favors cell survival under the additional stress of single cell plating in very low density for clonogenic assays; 3) and conversely, sustained inhibition of Src activity by PP1 and PP2 in long term mass culture in serum strongly limited rate of cell proliferation, which was restored by FGF2. Apparently, Src inhibition was sufficient to impair proliferation, whereas FGF2 mitogenic activity counteracted the anti-proliferative effect of Src inhibitor.

It is noteworthy that G0/G1-arrested Y1 cells maintained in serum-free media exhibited drastic reduction in basal levels of DNA synthesis upon treatment with PP1 and PP2, as shown by [^3^H]-thymidine pulse labeling and BrdU sustained labeling (Figures 6E and 6F, respectively). These observations suggest that the constitutive high levels of active Src underlie the deregulation of the control switch of G1 phase starter in Y1 cells. In addition, interestingly, G0/G1-arrested Y1 cells treated with Src inhibitors responded to FGF2 with unrestricted cell cycle progression through the G0/G1 → S → G2 transitions as well as cell division (Figure 6F). Therefore, the constitutive high levels of active Src seem to underlie both the deregulation of the G0/G1 → S transition and also the susceptibility of Y1 cells to FGF2’s irreversible blockage of G2 → M transition.

**Figure 7:**
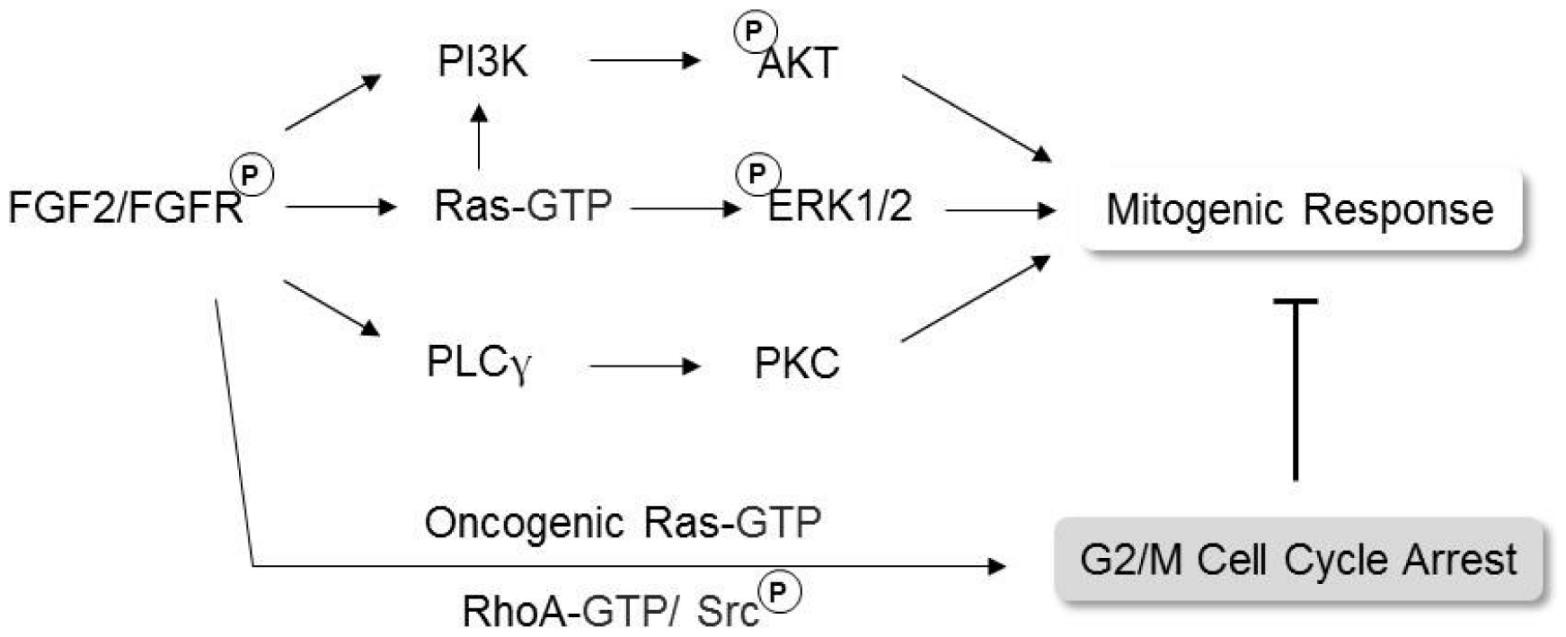
Proposed mechanism for the cytostatic effects of FGF2 in malignant cells transformed by Ras. FGF2 normally activates FGF receptors that trigger mitogenic responses. However, in Ras-transformed cells, FGF2 activates anti-proliferative mechanisms that are dependent on the Ras oncogene, active RhoA and Src tyrosine kinases, which ultimately trigger G2/M cell cycle arrest and senescence.

We recently reported that the inhibition of RhoA activity by C3 exoenzyme or ectopic expression of a dominant negative RhoA mutant abolished FGF2-induced morphological alterations and completely prevented FGF2’s cytostatic activity in both Y1 and 3T3^Ras^ cells [18]. The inhibition of RhoA activity blocks both migration and division of normal cells, but not malignant cells. Thus, K-Ras-dependent Y1 malignant cells regularly divide even when RhoA activation is blocked and migration is completely inhibited. The involvement of Rho-GTPases in oncogenesis is the subject of a sizable chunk of the cancer biology literature. Despite this extensive amount of scholarship, the actual role of Rho-GTPases in oncogenesis remains obscure. In particular, Src and RhoA-GTPase exhibit a complex interplay in oncogenesis. Initially, it was believed that Src-induced malignant transformation of fibroblasts was a cause of drastic reduction in RhoA-GTPase levels, mechanistically explaining the disappearance of actin stress fibers in Src-transformed fibroblasts [50]. However, Berdeaux and colleagues [51] used an *in situ* assay to show that RhoA-GTPase is highly active and necessary to maintain the malignant phenotype of Src-transformed fibroblasts. In addition, more recently, Lee and co-workers [52] showed that in normal cells, RhoA-GTPase regulation of focal adhesion dynamics is modulated downstream by Src promotion of ROCKII phosphorylation at Y722. Furthermore, another downstream effector of Rho-GTPases, mDia1, is also involved in the Src pathway. Tanji and colleagues [53] reported that primary cultures of embryonic fibroblasts derived from knockout mice deficient in mDia1 are resistant to transformation by v-Src. These observations imply that the Rho/mDia1 pathway mediates v-Src translocation to the cell periphery, disruption of actin stress fibers and formation of podosomes. Altogether, these results show that an intricate network comprised of Src kinases and Rho-GTPases underlies migration, morphological alterations, cytoskeletal dynamics and dysfunctional regulation of the cell cycle, all phenotypic traits of cancer cells. It is within this scenario that the Ras-dependent Y1 mouse malignant cell line is a useful experimental model. High chronic levels of K-Ras-GTP underlie the malignant phenotype of Y1 cells, which includes susceptibility to a RhoA-dependent FGF2’s cytostatic effect [18]. Thus, Y1 stable sublines harboring a dominant negative RhoA mutant displayed constituvely suppressed endogenous RhoA activity, proliferated at optimal rates in serum and was resistant to FGF2’s cytostatic effects [18]. Differently, Y1 cells treated with Src specific inhibitors proved to be resistant to cell cycle arrest by FGF2, but were not able to proliferate in serum (Figure 6A). Altogether, these results imply that in spite of RhoA or Src activity downregulation completely protect Y1 cells from FGF2’s cytostatic effects, Y1 sublines lacking RhoA activity proliferate well; while Src activity above a certain threshold seems to be imperative for parental Y1 cells proliferation. In conclusion, we tentatively suggest that, in K-Ras-driven malignant cells, the FGF2/FGFR signaling axis activates the pathway Src → RhoA to block the cell cycle G2 → M transition independently from the classical PI3K/Akt, Ras/ERK and PLCγ/PKC mitogenic pathways, as schematically depicted in Figure 7.

## Materials and Methods

### Cell Lines and Cell Culture

The Y1 murine adrenocortical carcinoma cell line [54] was obtained from ATCC in 1973. Y1 cells were grown at 37°C in a 5% CO_2_ atmosphere in Dulbecco’s modified Eagle’s medium (Invitrogen) supplemented with 10% fetal calf serum (CultiLab), 2 mM glutamine, 100 U/mL penicillin and 100 mg/mL streptomycin. The early passage Balb/c mouse embryo fibroblast cell line, Balb 3T3, clone A31, was obtained in 1984 from the laboratory of Dr. Charles D. Stiles (Dana-Farber Cancer Institute, Harvard Medical School, Boston, MA). The 3T3^Ras^ cell line (or 3T3-B61) was derived from Balb 3T3 fibroblasts, clone A31, transfected in our laboratory with a constitutively expressed H-Ras^V12^ oncoprotein [43]. 3T3^Ras^ cells were cultured in the same conditions as Y1, and the growth medium was supplemented with 0.1 mg/mL geneticin (G418; Invitrogen). Cells were passaged using 0.25% trypsin-EDTA (Invitrogen).

### G0/G1 Synchronization by Serum Starvation

Approximately 1.5×10^4^ cells/cm^2^ were plated in polystyrene plates (Corning). The next day, the medium was removed and replaced by fresh Dulbecco’s modified Eagle’s medium (without serum). After 48 hours of serum starvation, cells were stimulated with 10% fetal calf serum (Cultilab) or recombinant FGF2 (obtained in our laboratory).

### Clonogenic Assays and Growth Curves

For clonogenic assays, 50–250 cells/cm^2^ cells were plated in polystyrene plates (Corning). After incubation (10–14 days), colonies were fixed in 4% formaldehyde in phosphate-buffered saline (PBS), stained with 0.1% crystal violet and counted [55]. To analyze cell proliferation, 10^4^ cells/cm^2^ were plated in 35 mm dishes and counted daily for 4–7 days with changes in the medium every second day.

### Cellular Viability Assays

Cellular viability was analyzed in exponentially growing Y1 cells by the MTS assay (CellTiter 96® AQueous, Promega) and the plasma membrane integrity assay (CytoTox-One™, Promega) according to the manufacturer’s instructions.

### RNAi

To knockdown FGFR2 and FGFR3, pSUPER [56] and pX343 plasmids (genetic marker) [57] were co-transfected into Y1 parental cells at 50% confluency in a 60 mm-diameter plate using lipofectin reagent (Invitrogen). The short hairpin oligonucleotides were a kind gift from Dr. Ivan T. Rebustini and Dr. Matthew P. Hoffman, NIH, USA. Cells were selected and maintained with 0.4 mg/mL hygromycin B (Invitrogen). To knockdown FGFR1, siRNAs (ID 158530, 158531, 158532, Ambion), GAPDH (siRNA ID AM4624, Ambion), and a nonsilencing scrambled siRNA sequence were used (siRNA ID AM4635, Ambion) were transfected with siPORT Amine (Ambion). Knockdown efficiency was analyzed by semi-quantitative or quantitative RT-PCR.

### RNA Extraction and Reverse Transcription (RT)

Cells were harvested with Trizol. The RNA pellet was diluted in autoclaved bidistilled water and quantified by absorptiometry at 260 nm. The extracted RNA was treated with 1 U DNAse per µg RNA to eliminate contaminant DNA. Five microgram total RNA was reverse-transcribed into cDNA at 50°C for 90 min with 200 U transcriptase SuperScript III, 1X First-Strand Buffer, 5 mM Dithiothreitol, 500 nM of each dNTP, and 25 ng/µL (all reagents from Invitrogen).

### Polymerase Chain Reaction (PCR)

The template cDNA obtained from the reverse transcription were incubated with 250 U/µL Taq DNA Polymerase, 1X PCR buffer, 1.2 mM dNTPs mixture, 1.5 mM magnesium chloride (all reagents from Invitrogen), 0.5 µM primers forward and reverse (IDT) targeting FGFR1, FGFR2, FGFR3, FGFR4, FGFR5, and GAPDH. Glyceraldehyde-3-phosphate dehydrogenase (GAPDH) was used as an internal standard due to its relatively constant expression in studied cells. For each reverse transcription reaction, a negative control (RNA without reverse transcriptase) was included to verify the absence of DNA contamination. The samples were analyzed in 1,5–2.0% agarose gels.

### Quantitative Polymerase Chain Reaction

The template cDNA obtained from the reverse transcription were incubated with Sybr Green Mixture (Applied Biosystems) and 650 nM primers forward and reverse primers (IDT) targeting FGFR1, FGFR2, FGFR3, FGFR4, FGFR5, and HPRT1. The reaction was done on the thermocycler AB 5700 (Applied Biosystems). Hypoxanthine guanine phosphoribosyl transferase (HPRT1) was used as an internal standard due to its relatively constant expression in studied cells. The 2^−ΔΔ^Ct method was applied for data normalization and statistics analysis [58].

### Sequencing of RT-PCR Products

The PCR products were purified from agarose gels using the GFX kit (Amersham-Pharmacia Biotech). DYEnamic ET Dye Terminator Kit (Thermo Sequenase™ II DNA Polimerase, GE Healthcare) were used to sequence the PCR products.

### SDS-PAGE and Immunoblot

Cells were washed with cold PBS, harvested in a lysis buffer (pH 7.5) containing 1% NP-40, 150 mM sodium chloride, 50 mM Tris-Cl, 0.1% sodium dodecyl sulfate, 0.5% sodium deoxicholate, 1 mM sodium ortovanadate, 1 mM PMSF, 2 µg/mL aprotinin, 2 µg/mL pepstatin A and 2 µg/mL leupeptin, vortexed extensively and centrifuged at 12,000×g for 10 minutes at 4°C. Supernatant aliquots of proteins were resolved by sodium dodecyl sulfate-polyacrylamide gel electrophoresis (SDS-PAGE; 12% acrylamide/bis-acrylamide) and transferred onto Nylon membranes (Hybond C+, Amersham Biosciences). Membranes were blocked for 1 hour in TBS-T buffer (150 mM sodium chloride, 50 mM Tris [pH 8], and 0.1% Tween 20) containing 5% nonfat milk and were then incubated with antibodies recognizing AKT (#9272), phospho-AKT Ser473 (#9272), ERK1/2 (#9102), phospho-ERK1/2 Thr202/Tyr204 (#9101L), phospho-Src Tyr416 (#2101), total Src (#2110) (all from Cell Signaling), and Hprt (sc-20975) (Santa Cruz). After incubation with anti-IgG secondary antibodies (rabbit) conjugated to horseradish peroxidase (Amersham), immunoreactive signals were visualized by using enhanced chemiluminescence (Amersham).

### Cell Cycle Analysis by Flow Cytometry

After synchronization in G0/G1 by serum starvation, cells were treated with serum and/or FGF2 as well as 50 µM bromodeoxyuridine (BrdU) at time 0. After assorted experimental or control treatments, cells were trypsinized, washed with ice-cold PBS and fixed in ice-cold 75% ethanol in PBS for 20 minutes at 4°C. DNA was denaturated in 2 M HCl and 0.5% Tween-20 for 15 min. Cells were then washed sequentially with 0.1 M sodium tetraborate (pH 9.5) and ice-cold PBS. Cells were treated with 2 µg/mL anti-BrdU coupled to Alexa 488 in PBS (Invitrogen) for 30 minutes. After washing with ice-cold PBS, cells were treated with 10 µg/mL RNase A and stained with 50 µg/mL propidium iodide in PBS for 20 minutes before analysis in a flow cytometer (FACScalibur Flow Cytometer, BectonDickinson). The programs WinMDI V2.9 (The Scripps Research Institute, San Diego, CA), Cylchred V1.0.2 (CytonetUK, Cardiff University, UK) and FlowJo 7.5 (Treestar, Inc.; free trial) were used for data analysis. Light scattering was used to monitor cell size and granularity or internal complexity.

### Chemicals

The following chemical inhibitors were used: PD173074 (Sigma), Gö6983 (Sigma), LY294002 (Promega), Wortmannin (Sigma), U0126 (Promega), PD98059 (Sigma), PP1 and PP2 (Merck).

## Supporting Information

Figure S1 **Y1 cells express FGFR1, FGFR2, FGFR3 and FGFR5.** This figure shows the **s**equencing of PCR fragments harboring the splicing sites between IgIIIa and IgIIIc of FGFR1, FGFR2 and FGFR3 in Y1 cells.
(DOC)

Figure S2 **FGF2 delays S phase entry and irreversibly restrains cell division.** Flow cytometry histograms of DNA content in Y1 cells after FCS (10%) and FGF2 (10 ng/mL) treatment in G0/G1-starved cells begun at 0 h. Samples were taken at the indicated time points (12, 24, 36, 48 and 60 h after treatment). DNA content histograms (upper panels) and DNA/BrdU scatterplots (lower panels) of Y1 cells in the absence or presence of PD173074 (**A**, − PD173074; **B**,+PD173074, respectively). In the upper panels, quantification of G0/G1, S and G2/M phases was based on DNA content. (**C**) Quantification of G0/G1, S and G2/M phases based on DNA content versus BrdU labeling. Quantification of cell cycle phases, gated from the 2N to the 4N population only. Approximately 2 × 10^4^ cells were analyzed. SFM, serum-free medium.
(DOC)
