# Supplementary figures and images for: Fibroblast Growth Factor 2 Causes G2/M Cell Cycle Arrest in Ras-Driven Tumor Cells through a Src-Dependent Pathway
Source: PLoS One. 2013 Aug 26;8(8):e72582. doi: 10.1371/journal.pone.0072582 (PMC3753234; doi:10.1371/journal.pone.0072582)

**Figure S2**

**A**


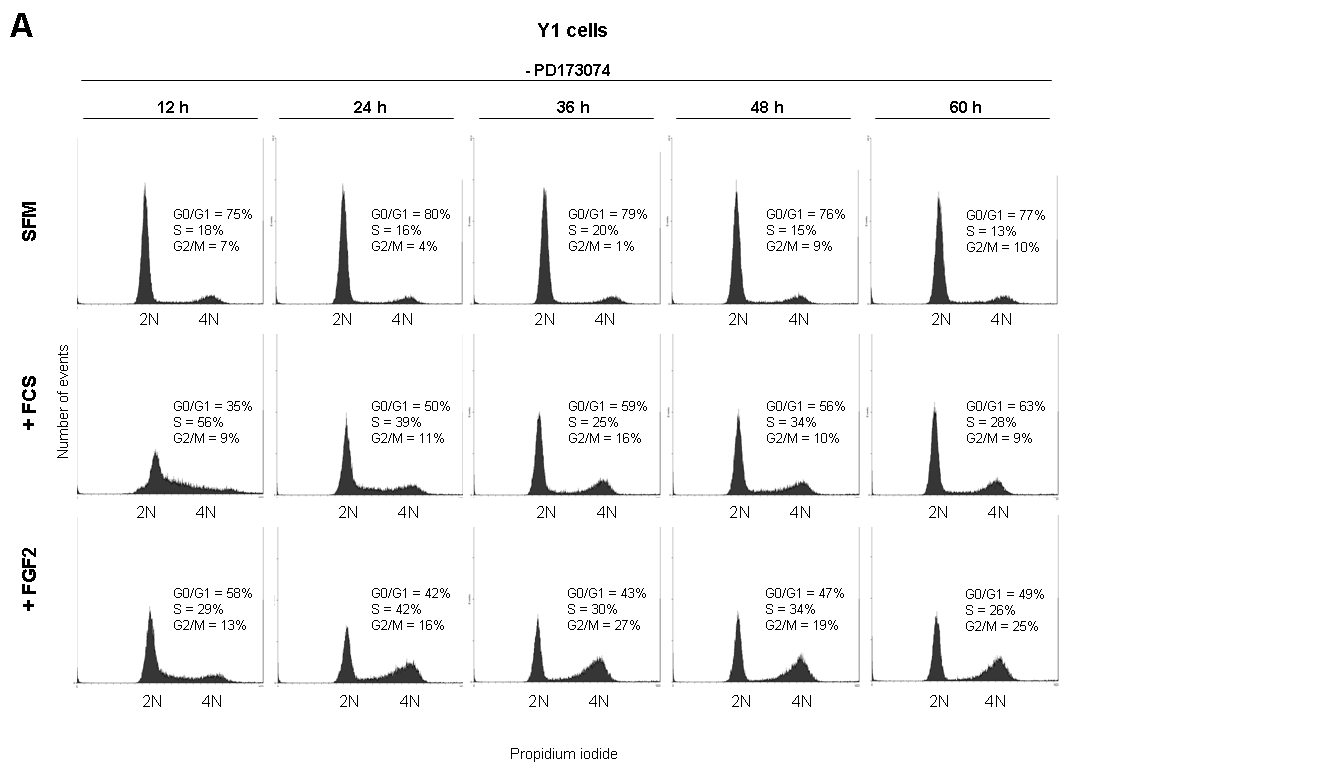

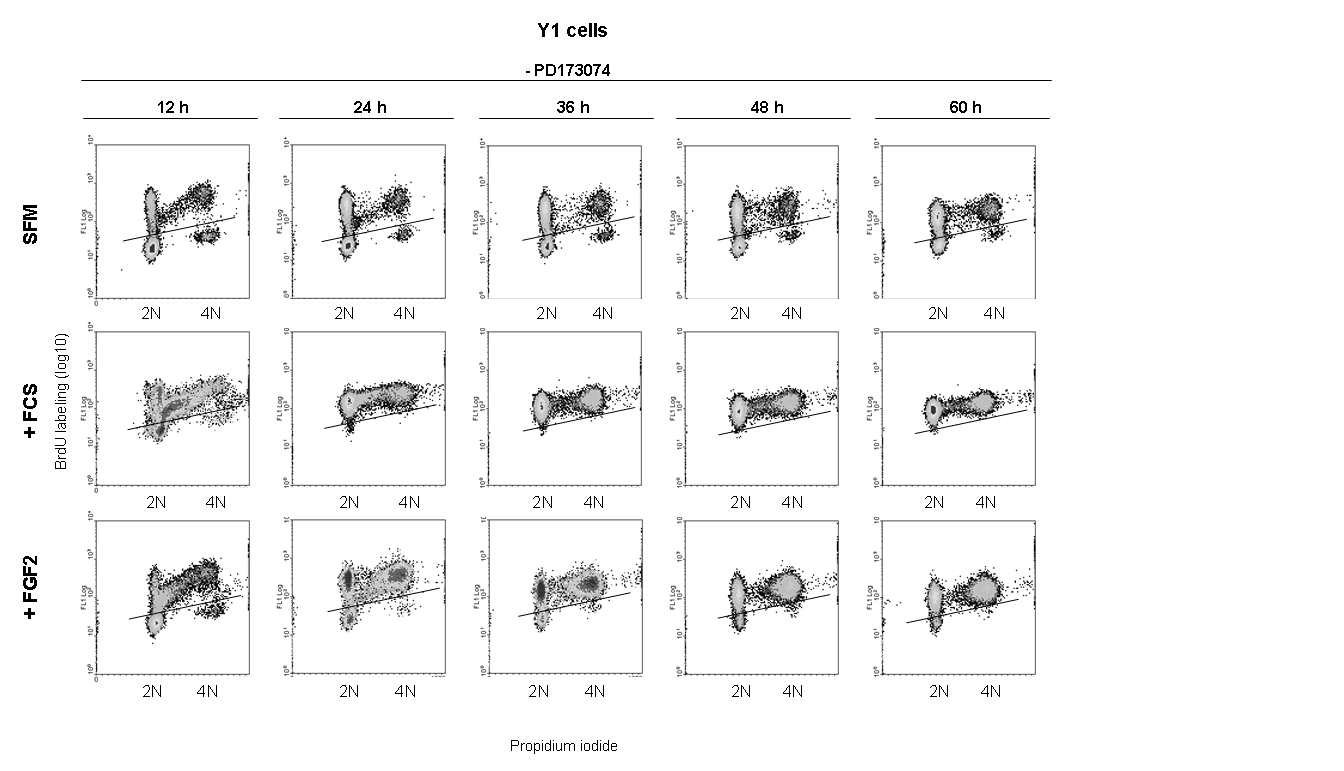


**B**


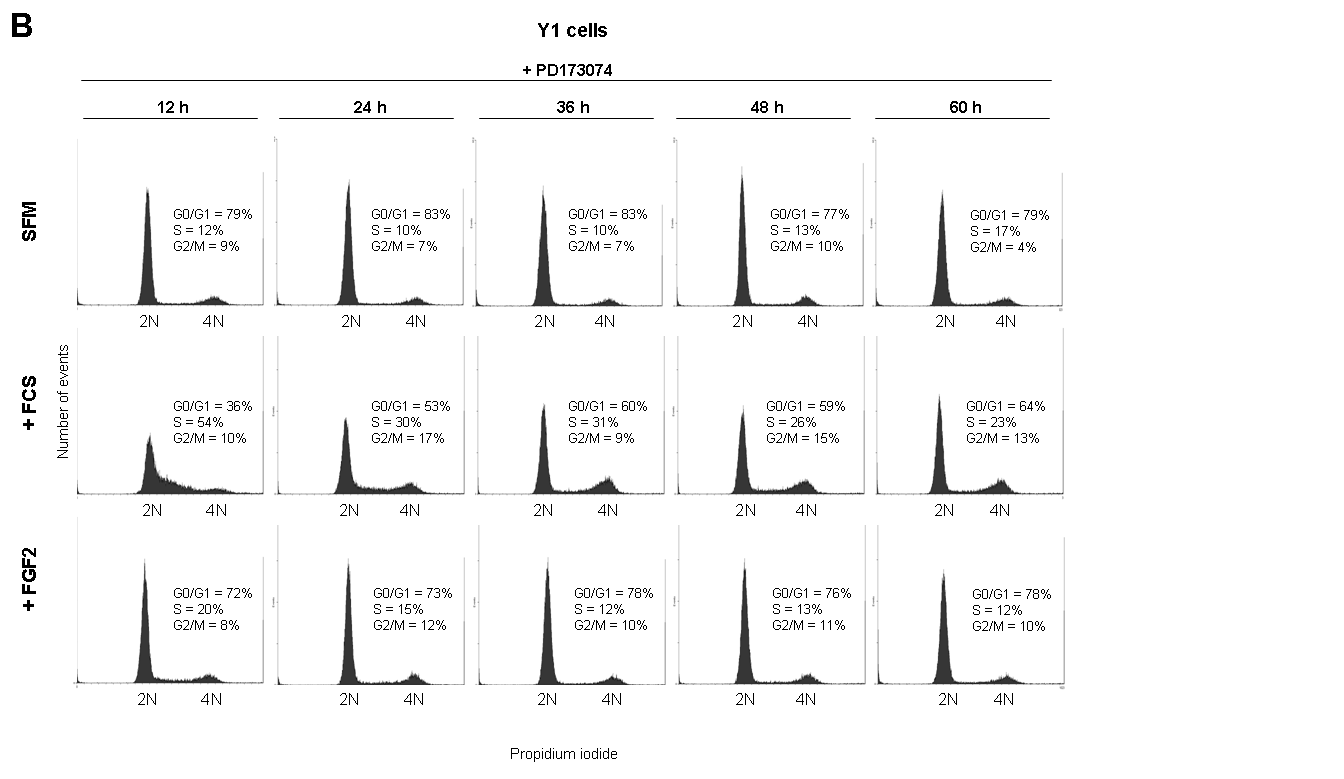

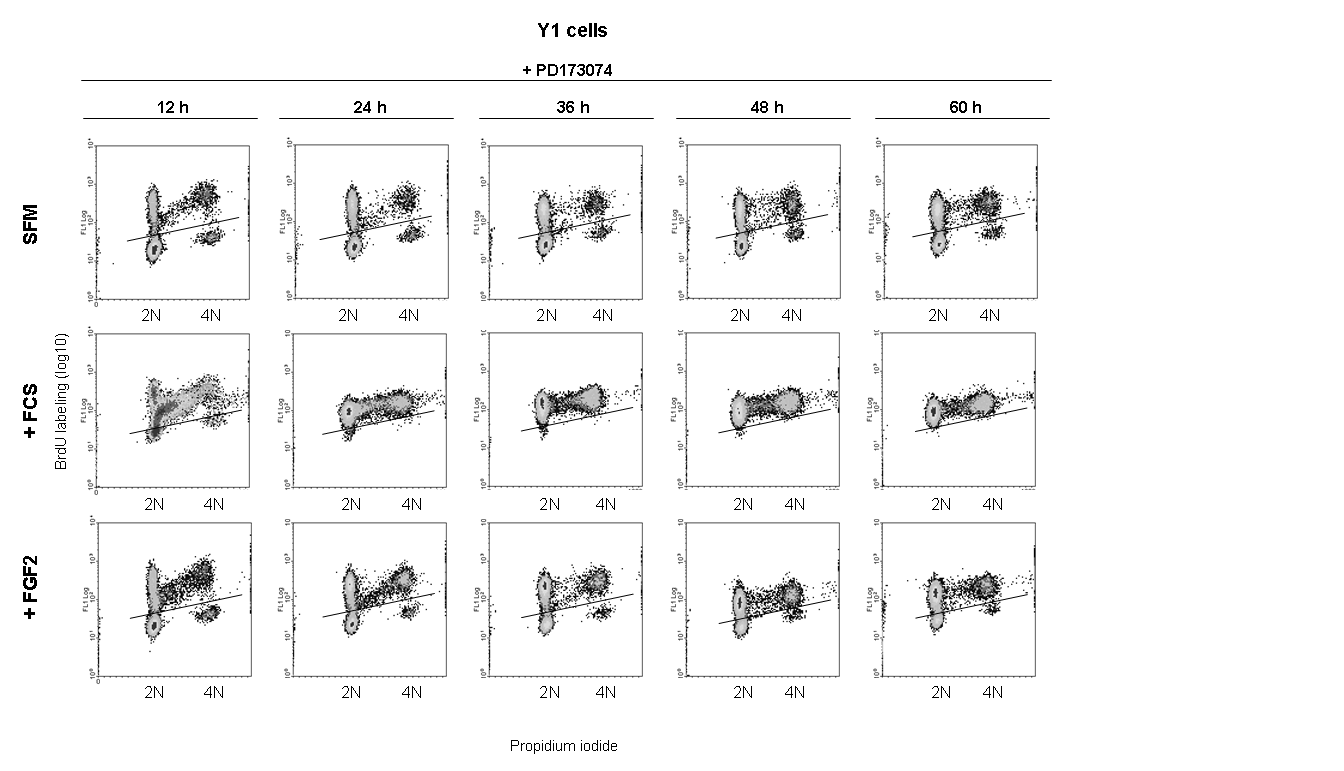


**C**

Supplement: Figure S2 — FGF2 delays S phase entry and irreversibly restrains cell division. Flow cytometry histograms of DNA content in Y1 cells after FCS (10%) and FGF2 (10 ng/mL) treatment in G0/G1-starved cells begun at 0 h. Samples were taken at the indicated time points (12, 24, 36, 48 and 60 h after treatment). DNA content histograms (upper panels) and DNA/BrdU scatterplots (lower panels) of Y1 cells in the absence or presence of PD173074 (A, − PD173074; B,+PD173074, respectively). In the upper panels, quantification of G0/G1, S and G2/M phases was based on DNA content. (C) Quantification of G0/G1, S and G2/M phases based on DNA content versus BrdU labeling. Quantification of cell cycle phases, gated from the 2N to the 4N population only. Approximately 2 × 10^4^ cells were analyzed. SFM, serum-free medium. (DOC) [file pone.0072582.s002.doc]
